# Society Proceedings

**Published:** 1912-07

**Authors:** 


### SOCIETY PROCEEDINGS

THE recent meeting of the A. M. A. at Atlantic City was most successful. The attendance at these meetings for the last few years has been as follows:

| | | | |
|---|---|---|---|
| 1900 Atlantic City | 2,019 | 1906 Boston | 4,722 |
| 1904 Atlantic City | 2,894 | 1908 Chicago | 6,446 |
| 1907 Atlantic City | 3,713 | 1910 St. Louis | 4,084 |
| 1909 Atlantic City | 3,273 | 1911 Los Angeles | 2,153 |
| 1912 Atlantic City | 3,600 | | |

The larger attendances are pretty obviously proportionate to the increment of the local profession. President Murphy, in his address to the house of delegates, called special attention to the need of increasing the membership, citing the following statistics:

| | | | |
|---|---|---|---|
| May 1, 1908 | 31,343 | 1911 | 33,960 |
| 1909 | 33,935 | 1912 | 34,283 |
| 1910 | 34,176 | | |

Roughly speaking, therefore, the normal attendance at the annual meetings, barring allowance for local increase and diminution due to high average distance, is slightly over 10%. Between a quarter and a fifth of all physicians in the country belong to the A. M. A.

The next meeting will take place in Minneapolis under the presidency of Dr. A. Jacobi of New York, with Dr. John A. Witherspoon of Nashville as president-elect. Our own territory is represented by Dr. John L. Heffron of Syracuse, as second vice-president.

The relegation of all business to the house of delegates and the perfecting, with increased experience of the means of registering, providing amusement, scientific and commercial exhibits, &c., has removed many confusing factors from these great medical gatherings. Some problems are left unsolved and are, perhaps insoluble. One of these is to provide halls of convenient location and size, of such acoustic properties that comparatively untrained speakers may be readily heard, and with such ventilation that large audiences may be comfortable. Another is to secure proper attention of audiences for several days of assiduous attendance, and still allow time for social gatherings, discussion of business and professional matters by individuals meeting by chance or by appointment, careful study of the commercial, scientific and other educational exhibits, &c.

A minor point to be considered is the advisability of continuing the annual ball. If this institution is to be maintained, it ought to be begun at a seasonable hour, considering the fatigue of the attendants and the serious business of the next day; the orchestra should be instructed that it is not playing for the gambols of a high school dance; there should be some authority to bar the turkey trot and the hosiery exhibits that marred the dignity of the recent one, and that undoubtedly were attributable to uninvited guests.

The writer's first experience with the meetings of the A. M. A. was at Nashville in 1890. Not to mention the very marked change in organization, exclusion of politics and business from the general sessions and the establishment of a policy of strong central government, one who has attended these meetings even occasionally, has had the opportunity to note a remarkable evolution. If we remember rightly, it was at the Columbus meeting in 1899

that popular sentiment developed that there was to be no more second-grade work by first-grade men; no more publication and, if possible, no more reading of inferior papers by anyone; no more interruption of section programs by celebrities who ran from place to place, dropping words of wisdom as they ran. In other words, the quality of the scientific work suddenly rose and, for a number of years, barring inevitable exceptions and perhaps the transactions of closely limited, technical societies, the transactions of the A. M. A. are second in scientific value to those of no organization in the medical world.

Even in externals, the men—and the accompanying women—seen at these great national conventions, have improved. The benefits of mutual attrition have been manifest in various ways. There is developing an esprit de corps, frankly, perhaps harshly critical at times, but loyal at heart and making for professional solidarity.

---

The Buffalo Society of Sanitary and Moral Prophylaxis met at the Genesee Hotel, June 17. The investigating committee reported an estimate of 4800 persons afflicted with venereal disease at any one time, in the city (a very conservative estimate if we include chronic gonorrhoea and tertiary syphilis). The work of this society can be furthered by taking membership at one dollar a year, remitting to Dr. Robert W. Hinds, Secretary.

---

At the annual meeting of the Buffalo Academy of Medicine, June 11, reports of the year's work were received, indicating an average attendance of 75—100, at each of the eight meetings of each of the four sections. About half of the programs were furnished by out-of-town men. Exact figures are impossible, on account of the failure of one of the sections to report. The retiring president, Dr. S. Y. Howell, gave a stirring address with various ethical problems confronting the profession. Dr. Wm. Scott Renner was elected president for the coming year. The meeting ended with a collation.

---

The Rochester Pathological Society held the annual picnic at the Island Cottage on the lake shore, June 13, 1912. About 50 were present. Dr. Wynn B. Palmer made his address as retiring president on "Ethical Proprietaries, Frauds and Nostrums," hitting straight from the shoulder. The new president is Dr. George A. Marion.

---

The Rochester Blackwell's Medical Society of women physicians also held a picinc upon the same date, June 13, 1912, at which Dr. Li told of her experiences in China. Dr. Mary A. Nickerson presided.

---

The Monroe County Sanitary Association met June 18, 1912, to discuss vaccination and other sanitary measures. Dr. H. J. Mann, of Brockport presided.

---

MEDICAL DEPARTMENT OF THE UNIVERSITY OF BUFFALO—37th Annual Meeting of the Alumni—Commencement Exercises.

Tuesday, May 28, reunions of classes ending in 2 and 7, were held.

Wednesday morning, Dr. G. H. A. Clowes of the N. Y. State Institute for Malignant Diseases, described the Serum Reaction in Gonorrhea and Dr. E. R. Larned of Detroit, spoke on Phylacogens. At noon, the visiting alumni were given an auto ride, followed by a luncheon in the college library. In the afternoon, Dr. Samuel G. Grant, Professor of Rectal Diseases in the N. Y. P. G. Medical School and Hospital, gave a lecture and clinic. Thursday morning, there was a symposium on Syphilis, Dr. G. Howard White of Baltimore discussing serology; Dr. Howard Fox of the N. Y. Skin and Cancer Hospital illustrating the skin manifestations with lantern slides; Dr. Charles F. Hoover, of Cleveland speaking of the visceral manifestations. After luncheon in the college library as guests of Drs. H. C. Buswell and E. J. Meyer, Dr. Hoover gave a clinic on aneurysm. The latter part of both Wednesday and Thursday afternoons was devoted to the Harrington lectures, this year by Dr. Ludwig Kektoen of Chicago, on Immunity in Relation to Surgery. (These will be published in this journal) The smoker, tendered by the Dean and Faculty, was held Wednesday evening.

The annual alumni dinner was held Thursday at the Statler, Dr. Peter W. Van Peyma acting as toast master and displaying as keen a wit as he has demonstrated a seriousness in technical matters. Under the leadership of Dr. Busch, many songs were sung. Of the various toasts, allusion can be made here, only to the frank discussion of the problems of the college and the eloquent appeal for alumni support, by Dr. Park. Nearly 200 alumni were registered at the meeting and of these, about 75 were from outside the city.

The commencement exercises were held Friday morning in the Teck Theatre, the address being given by Senator George B. Burd. Graduates in medicine numbered 40; in pharmacy 40; in analytic chemistry 7; in law 37 and in dentistry 30. Following the exercises, the graduates and alumni were entertained by the faculty at luncheon at the University Club. The graduates in medicine were as follows:

| | |
|---|---|
| Abraham Highman Aaron | Joseph Edward Maryanski |
| *Nathaniel Alpert | George Peter Michel, *Ph.B., A.M.* |
| Peter Joseph Barone | John Robert Monihan |
| Lawrence Melvin Belzer | Jerome Aloysius Murphy |
| Theron Blain Bond | Martin Francis Nolan |
| George Robert Colline | Joseph Aloisius Nowicki |
| LeGrand Augustus Damon | Faye Horace Palmer |
| John Tom Donovan | Frank Nelson Potts |
| Alva Garfield Dunbar | Edward Emmett Powers |
| Henry DeWitt Duryea | Grover Lee Priess |
| Frank Macbeth Ende | Leo James Rozan |
| Harold Henry Fox | Floyd George Schuler |
| Milton Harry Goldberg | Elmer George Steel |
| Harry Carl Guess | *Clare Nelson Shumway |
| Leon Couch Hamilton | George Romain Stalter |
| John Arthur Kneller | Walter Cyril Steele |
| Irving Nelson Kohler, *A. B.* | Nelson Wilbert Strohm |
| John Joseph Kohlhas | Horace Rowe Taylor |
| William John Lavelle | Worthington Charles Ward |
| Oliver Angus McCall | Orton E. White |

*Diploma withheld on account of deficiency in time.

---

On June 6th at Atlantic City, during the meeting of the American Medical Associtio and following a symposium on anesthesia, the National Society of Anesthetists was organized. Prof. Yandel Henderson of Yale, chairman of the commission on anesthesia of the A. M. A. occupying the chair, those essembled for the symposium acting as a committee of the whole, proceeded to organization and elected the following officers for the year 1912-13.

President James T. Gwathmey of New York, Vice Presidents F. H. McMeechen of Cincinnati, Charles K. Teter of Cleveland, Yandel Henderson of New Haven. Secretary William C. Woolsey, 88 Lafayette Ave., Brooklyn. Treasurer Harold A. Sanders of Brooklyn.

The constitution and by-laws were ordered to be drawn by the executive committee and submitted to the Society at its next meeting for adoption; all names submitted for membership, if qualified in the estimation of the executive committee, shall be considered as charter members if presented within a period of sixty days and accompanied by the levied dues of three dollars.

The National Society of Anesthetists in this notice calls all those who are actively interested in this work to join its ranks and assist in developing the subject of anasthesia to greater perfection and more uniform safety.

WILLIAM C. WOOLSEY, *Secty.*

June 10th, 1912.

---

### Synopsis of Minutes of Meeting of Medical Society of the County of Erie, June 17th, 1912.

MEETING held in the Buffalo Library Building, called to order, at 9 p. m., by 2nd Vice-President Dr. John V. Woodruff.

Minutes of previous meeting and Council meetings of May 6th and June 12th read and adopted.

Resignations of Drs. George W. Schaefer and G. K. Lester of Blasdell, N. Y., were accepted.

Dr. Bonnar, Chairman of the Board of Censors, reported a barber of Lackawanna N. Y., had been fined $25.00 for cupping.

Resignation of Mr. Charles A. Doane as counsel for the society was accepted with regret and a vote of thanks tendered him for his able services in the past.

A special committee, of which Dr. Floyd S. Crego was chairman, reported regarding the need of a Psychopathic Ward or Hospital and recommended the construction of such a hospital on the West Farms site, which has been selected by the city of Buffalo for the construction of a Municipal General Hospital, such recommendation being adopted by the society.

Dr. Henry R. Hopkins was elected an honorary Vice-President to the 4th International Congress on School Hygiene to be held in Buffalo, August 25th to 30th, 1913, also as delegate to said Congress from the Medical Society of the County of Erie.

The following papers were presented and discussed:

Symposium—Conservation of Vision, led by F. Park Lewis; Prevention of Ophthalmia Neomatorum, Dr. Arthur G. Bennett; Treatment of Ophthalmia Neonatorum, Dr. Lee Masten Francis.

---

The Annual meeting of the **Medical Alumni of Syracuse University** was held at the Medical College, Monday afternoon, June 10th. Dr. William T. Councilman, Professor of Pathology at Harvard, addressed the meeting on "Some Personal Recollections as a Teacher of Medicine." Dinner followed at the Onondaga, which was attended by nearly a hundred alumni. Dean Heffran acted as toastmaster and called upon Dr. Councilman, Commissioner Porter, of the State Board of Health, and Lawrence Sisson for after-dinner speeches.

